# Predictors of stroke volume improvement with AV-optimized conduction system pacing in patients with AV dromotropathy

**DOI:** 10.1093/eschf/xvag060

**Published:** 2026-02-19

**Authors:** Anja Zupan Mežnar, Tadej Žlahtič, Miha Mrak, Maja Ivanovski, David Žižek

**Affiliations:** Faculty of Medicine, University of Ljubljana, Vrazov trg 2, 1000 Ljubljana, Slovenia; Department of Cardiology, University Medical Centre Ljubljana, Zaloška 2, 1000 Ljubljana, Slovenia; Department of Cardiology, University Medical Centre Ljubljana, Zaloška 2, 1000 Ljubljana, Slovenia; Faculty of Medicine, University of Ljubljana, Vrazov trg 2, 1000 Ljubljana, Slovenia; Department of Cardiology, University Medical Centre Ljubljana, Zaloška 2, 1000 Ljubljana, Slovenia; Department of Cardiology, University Medical Centre Ljubljana, Zaloška 2, 1000 Ljubljana, Slovenia; Faculty of Medicine, University of Ljubljana, Vrazov trg 2, 1000 Ljubljana, Slovenia; Department of Cardiology, University Medical Centre Ljubljana, Zaloška 2, 1000 Ljubljana, Slovenia

**Keywords:** Atrioventricular dyssynchrony, Atrioventricular conduction block, Conduction system pacing, Atrioventricular optimization

## Abstract

**Aims:**

Patients with first-degree atrioventricular (AV) block and mechanical AV dyssynchrony can present with heart failure (HF)-like symptoms. AV-optimized conduction system pacing (CSP) can improve haemodynamics and symptoms, but selection criteria remain uncertain. We aimed to identify electrocardiographic and echocardiographic predictors of an acute haemodynamic response to AV-optimized CSP in symptomatic first-degree AV block.

**Methods and Results:**

Nineteen patients (mean age 60.5 ± 21.1 years; 37% female) with symptomatic first-degree AV block underwent baseline electrocardiography and echocardiography followed by AV-optimized conduction system pacing and repeat echocardiographic assessment. Electrocardiographic parameters (PR interval, P wave duration/PR interval ratio) and echocardiographic indices (E/A wave confluence, A-Q interval, and DFT/RR ratio) were tested for association with change in left ventricular stroke volume (LVSV).

The mean PR interval was 395 ± 61 ms, the mean A-Q interval 155 ± 65 ms, and the mean DFT/RR ratio 0.34 ± 0.1. E/A wave confluence was present in 15 patients (79%). AV-optimized pacing increased LVSV by 7.8 ± 3.9 ml, corresponding to an 11.8 ± 5.7% relative increase (*P* < .01). Echocardiographic parameters were associated with LVSV response, including A-Q interval (r = 0.63, *P* = .004), DFT/RR ratio (r = −0.59, *P* = .008), and E/A wave confluence (r = 0.57, *P* = .01). Electrocardiographic parameters were not associated with LVSV change.

**Conclusions:**

Echocardiographically assessed mechanical AV dyssynchrony, rather than electrocardiographic parameters, is associated with an acute haemodynamic response to pacing. Echocardiographic evaluation may help identify patients with prolonged PR interval who could benefit from AV-optimized CSP.

## Background

Patients with first-degree atrioventricular (AV) block and AV dyssynchrony may experience symptoms resembling heart failure (HF) owing to suboptimal ventricular filling and reduced left ventricular stroke volume (LVSV).^1^ Although AV-optimized conduction system pacing (CSP) has been shown to improve symptoms and LVSV in these patients, irrespective of baseline ejection fraction,^2,3^ patient selection remains challenging. Notably, secondary analyses from the HOPE-HF trial suggested that the acute haemodynamic response to pacing is the most reliable predictor of clinical benefit, rather than baseline PR interval or echocardiographic E/A wave fusion.^4^ However, this approach requires an invasive procedure before determining potential benefit, limiting its broader clinical adoption.

We hypothesized that echocardiographic assessment of AV dyssynchrony could help identify patients who are most likely to benefit from AV-optimized pacing.

## Aims

The aim of this study was to investigate electrocardiographic and echocardiographic parameters that predict a positive haemodynamic response to AV-optimized CSP in patients with symptomatic first-degree AV block.

## Methods

Between February 2020 and September 2022, patients screened for a single-centre, randomized, crossover AV dromotropathy study underwent prospectively planned baseline assessments, including PR interval measurement and echocardiography. The patient flow has been described previously.^2^ This subanalysis includes individuals who received a CSP device (predominantly His bundle pacing) and had follow-up echocardiograms after AV-delay optimization. We included all 17 patients enrolled in the original randomized study and two additional patients who were excluded from that trial due to reduced left ventricular ejection fraction (total *n* = 19). No other excluded patients underwent conduction system pacing. Exclusion criteria were: no symptoms, no echocardiographic AV dyssynchrony, PR interval shortening during exercise testing, pre-existing cardiac device, or a wide QRS complex. Electrocardiographic parameters (PR interval, P wave duration/PR interval ratio) and echocardiographic parameters (E/A wave confluence, A-Q interval [time interval between the end of A wave and the beginning of the QRS complex], and DFT/RR ratio [diastolic filling time/R wave to R wave interval ratio], all measured from transmitral pulsed-wave Doppler with the sample volume placed at the mitral annulus level) were tested for association with change in LVSV after pacing.^5^ Stroke volume was calculated from left ventricular outflow tract diameter and pulsed-wave Doppler velocity–time integral, obtained from apical five- and three-chamber views and averaged over at least five cardiac cycles. The values were cross-checked against the stroke volume obtained from the biplane Simpson's method. The echocardiographic acquisition and analysis protocol, including the AV-delay optimization procedure, has been described in detail previously.^2^ Linear regression analysis was performed using SPSS v. 29.0.

## Results

Nineteen patients with complete datasets were analysed. The mean age was 60.5 ± 21.1 years, 37% were female. All patients had marked first-degree AV block (mean PR interval 395 ± 61 ms), measured at a mean heart rate of 63 ± 8 bpm, and echocardiographic evidence of AV dyssynchrony. E/A wave confluence was observed in 15 patients (79%). The mean A-Q interval was 155 ± 65 ms, and the mean DFT/RR ratio was 0.34 ± 0.1. AV optimized conduction system pacing increased LVSV by 7.8 ± 3.9 ml, corresponding to an 11.8 ± 5.7% relative increase. Left ventricular ejection fraction did not differ significantly before and after pacing (63.2 ± 6.4% vs. 64.7 ± 6.7% with AV-optimized pacing).

**Figure 1:**
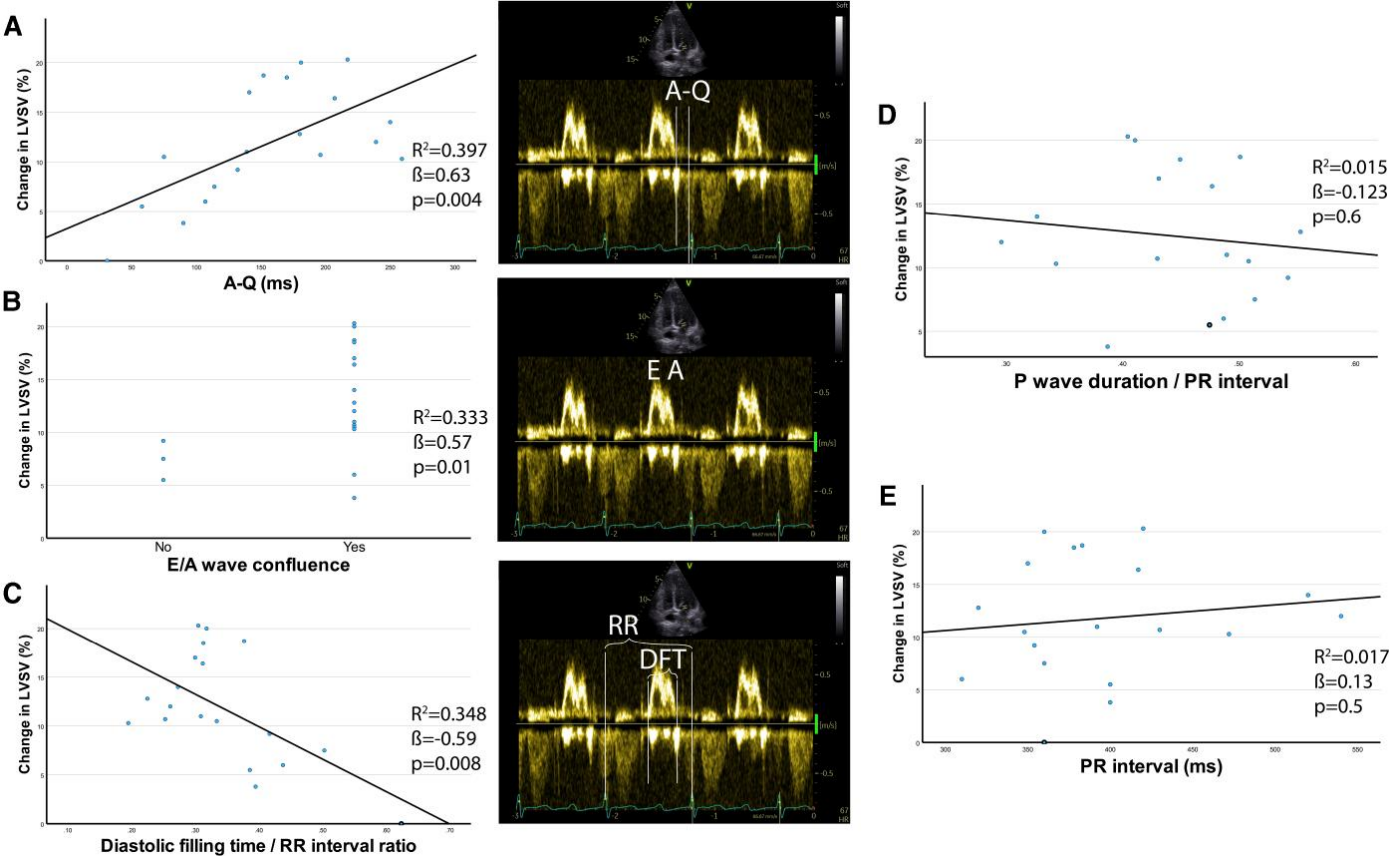
Linear regression analysis of electrocardiographic and echocardiographic parameters associated with relative left ventricular stroke volume (LVSV) increase. Panels A–C display echocardiographic parameters derived from transmitral Doppler flow analysis, each showing a significant association with relative LVSV increase after AV-optimized conduction system pacing: (A) A-Q interval, defined as the time from the end of the A wave to QRS onset, reflecting atrioventricular mechanical delay (*R*^2^ = 0.397, β = 0.63, *P* = .004); (B) E/A wave confluence, representing overlap or fusion of early (E) and late (A) diastolic filling waves (*R*^2^ = 0.333, β = 0.57, *P* = .01); (C) diastolic filling time/RR interval (DFT/RR) ratio, indexing diastolic filling duration to cardiac cycle length (*R*^2^ = 0.348, β = −0.59, *P* = .008). Panels D and E show electrocardiographic parameters with no significant association with LVSV change: (D) *P* wave/PR interval ratio (*R*^2^ = 0.015, β = 0.12, *P* = .6); (E) PR interval (*R*^2^ = 0.017, β = 0.13, *P* = .5)

Linear regression analysis showed that electrocardiographic parameters were not predictive of LVSV response to CSP. In contrast, echocardiographic parameters, particularly A-Q interval and DFT/RR, were significantly associated with the relative increase in LVSV. E/A wave confluence demonstrated a moderate positive correlation (*Figure 1*).

## Conclusions

Echocardiographically assessed mechanical AV dyssynchrony, rather than electrocardiographic parameters, appears to be the key determinant of the haemodynamic response to pacing therapy. Consistent with HOPE-HF trial subanalysis, neither the PR interval nor the P wave/PR interval ratio reliably predicted benefit, reflecting that these measures capture electrical rather than mechanical aspects of AV dyssynchrony.^4^

In contrast, echocardiographic measures such as the A-Q interval, DFT/RR ratio, and E/A wave confluence demonstrated stronger associations with LVSV increase following AV-optimized CSP. The positive correlation between the A-Q interval and LVSV improvement suggests that a prolonged delay between atrial contraction and ventricular activation may identify patients most likely to benefit from AV-optimized CSP. Moreover, the inverse relationship between the DFT/RR ratio and LVSV increase highlights the importance of optimizing diastolic filling relative to the cardiac cycle. In this analysis, which included patients with markedly prolonged PR intervals, E/A wave confluence demonstrated only a moderate positive correlation. It is therefore unsurprising that in the HOPE-HF subanalysis, where the mean PR interval was 249 ± 59.2 ms, there was no observed interaction between E/A wave confluence and the study endpoints.^4^

These findings suggest that echocardiographic assessment—particularly the A-Q interval and DFT/RR ratio—may assist in identifying patients with prolonged PR interval who could derive haemodynamic benefit from AV-optimized CSP. These results should be considered hypothesis-generating, and larger prospective studies are required to validate these parameters and determine whether their incorporation into patient selection and pacing algorithms improves clinical outcomes.

## Declarations

### Data Availability

The data underlying this article will be shared on reasonable request to the corresponding author.
